# Molecular Diagnostic of Solid Tumor Using a Next Generation Sequencing Custom-Designed Multi-Gene Panel

**DOI:** 10.3390/diagnostics10040250

**Published:** 2020-04-23

**Authors:** Dario de Biase, Giorgia Acquaviva, Michela Visani, Viviana Sanza, Chiara M. Argento, Antonio De Leo, Thais Maloberti, Annalisa Pession, Giovanni Tallini

**Affiliations:** 1 Department of Pharmacy and Biotechnology, Molecular Diagnostic Unit, University of Bologna, viale Ercolani 4/2, 40138 Bologna, Italy; dario.debiase@unibo.it (D.d.B.); chiaramaria.argento@unitn.it (C.M.A.); thais.maloberti@studio.unibo.it (T.M.); annalisa.pession@unibo.it (A.P.); 2 Department of Medicine (Dipartimento di Medicina Specialistica, Diagnostica e Sperimentale), Molecular Diagnostic Unit, University of Bologna, Azienda USL di Bologna, viale Ercolani 4/2, 40138 Bologna, Italy; giorgia.acquaviva3@unibo.it (G.A.); viviana.sanza@ausl.bologna.it (V.S.); antonio.deleo@unibo.it (A.D.L.); giovanni.tallini@unibo.it (G.T.)

**Keywords:** next generation sequencing, multi-gene custom panel, solid tumor, mutational analysis

## Abstract

Next generation sequencing (NGS) allows parallel sequencing of multiple genes at a very high depth of coverage. The need to analyze a variety of targets for diagnostic/prognostic/predictive purposes requires multi-gene characterization. Multi-gene panels are becoming standard approaches for the molecular analysis of solid lesions. We report a custom-designed 128 multi-gene panel engineered to cover the relevant targets in 22 oncogene/oncosuppressor genes for the analysis of the solid tumors most frequently subjected to routine genotyping. A total of 1695 solid tumors were analyzed for panel validation. The analytical sensitivity is 5%. Analytical validation: (i) Accuracy: sequencing results obtained using the multi-gene panel are concordant using two different NGS platforms and single-gene approach sequencing (100% of 83 cases); (ii) Precision: consistent results are obtained in the samples analyzed twice with the same platform (100% of 20 cases). Clinical validation: the frequency of mutations identified in different tumor types is consistent with the published literature. This custom-designed multi-gene panel allows to analyze with high sensitivity and throughput 22 oncogenes/oncosuppressor genes involved in diagnostic/prognostic/predictive characterization of central nervous system tumors, non-small-cell lung carcinomas, colorectal carcinomas, thyroid nodules, pancreatic lesions, melanoma, oral squamous carcinomas and gastrointestinal stromal tumors.

## 1. Introduction

Next generation sequencing (NGS) allows parallel sequencing of multiple genes at a very high depth of coverage. Considering the continuous discovery of molecules as a putative target or responsible for treatment resistance mechanisms, the application of a traditional single-gene approach is becoming problematic. Furthermore, precision medicine increasingly requires multi-gene characterization and for this reason the introduction of multi-gene panels is becoming crucial for the molecular analysis of solid lesions. Many multi-gene panels have become commercially available. However, these panels are usually designed for specific tumors or genes (e.g., QIAseq Human Colorectal Cancer Panel, CleanPlex^®^ BRCA1 & BRCA2 Panel; Oncomine BRCA Research Assay) and/or include a high number of targets (e.g., Oncomine Focus and Comprehensive Assays, GeneRead QIAact AIT DNA UMI Panel, Myriapod^®^ NGS 56G Onco panel, SOPHiA Solid Tumor Solution).

The abundance of targets leads to the routine sequencing of many markers that are of limited clinical relevance in a given tumor, reducing the total number of samples that can be analyzed in a single sequencing run and thus increasing the cost per sample. Developing custom multi-gene panels allows a rational selection of targets according to the needs of the biomedical community serviced by the molecular laboratory. This leads to the optimization of the number of specimens that can be analyzed in a single run to reduce costs.

The aim of the present work is to report a custom-designed multi-gene panel that allows for analyzing in the same NGS run most of the clinically relevant mutations in solid tumor types that represent the bulk of those tumors that need to be genotyped in most medical centers, i.e., central nervous system (CNS) tumors, non-small cell lung carcinomas (NSCLCs), colorectal carcinomas (CRCs), thyroid nodules, melanomas, gastrointestinal stromal tumors (GISTs), and squamous oral carcinomas (SOCs).

## 2. Material and Methods

### 2.1. Case Selection

A total of 1715 clinical samples were submitted for genotyping from January 2017 to December 2018 following a request of the caring clinician. Written informed consent for mutational analysis was obtained from all patients. Information regarding the human material was managed using anonymous codes and all samples were handled in compliance with the Helsinki Declaration. Follow-up information was not used for this study.

Samples were obtained from formalin-fixed and paraffin embedded tissues (FFPE), cytological smears or cytological fluid specimens. Twenty of 1715 (1.2%) samples could not be analyzed because they were pre-analytically inadequate for molecular analysis due to a small number of cells (i.e., less than 100) or to a low percentage of neoplastic cells (i.e., neoplastic cells/total number of cells below 10%) in the material submitted for molecular analysis. The remaining 1695 specimens were genotyped and are the object of this study.

**Figure 1:**
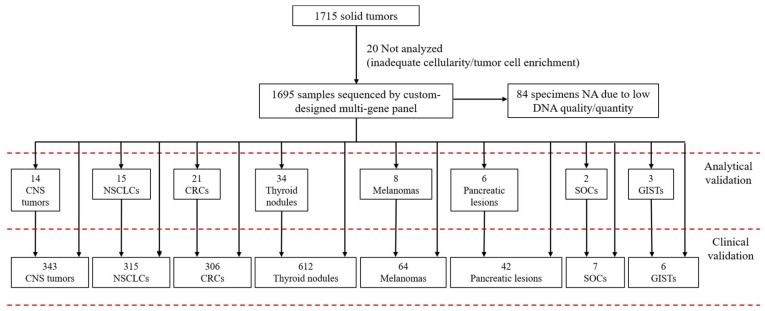
Cases analyzed by the custom-designed multi-gene panel. CNS: central nervous system/intracranial; NSCLCs: non-small cell lung carcinomas; CRCs: colorectal carcinomas; SOCs: squamous oral carcinomas; GISTs: gastrointestinal stromal tumors; NA not assessable: no amplifiable DNA.

Specimens included primary or metastatic lesions: 343 CNS/intracranial tumors, 315 NSCLCs, 306 CRCs, 612 thyroid nodules (pre-operative material), 64 melanomas, 42 pancreatic lesions (pre-operative material), 7 SOCs, and 6 GISTs (Figure 1).

### 2.2. Custom-Designed Multi-Gene Panel

The samples were analyzed using a panel customized for specific genomic regions including 128 amplicons (dual strand) for a total of 256 amplicons (15.04 kb; median amplicons size: 130 bp). The panel includes 58 target regions in the following 22 genes (human reference sequence hg19/GRCh37): *BRAF* (exon 15), *c-Kit* (exons 8, 9, 11, 13, 17), *CTNNB1* (exon 3), *EGFR* (exons 12, 18, 19, 20, 21), *EIF1AX* (exons 1, 2), *GNAS* (exons 8, 9), *H3F3A* (exon 1), *HRAS* (exons 2, 3), *IDH1* (exon 4), *IDH2* (exon 4), *KRAS* (exons 2, 3, 4), *MED12* (exon 2), *MET* (exon 2, 14), *NRAS* (exons 2, 3, 4), *PDGFRα* (exons 12, 14, 18), *PIK3CA* (exons 10, 21), *PTEN* (exon 5), *RET* (exons 5, 8, 10, 11, 13, 15, 16), *RNF43* (exons 2, 8), *SMAD4* (exons 6, 9, 10, 11, 12), *TERT* (promoter region, g.1295141–g.1295471), and *TP53* (exons 4, 5, 6, 7, 8, 9).

The genes evaluated depend on clinical guidelines and on the specific needs for treatment and diagnostic purposes as defined in the integrated care pathways for each tumor type of a given medical center (see “Results” section). To this point, mutations considered “pathogenic” were evaluated according to data reported in well-established mutation databases (e.g., COSMIC database [1], ClinVar https://www.ncbi.nlm.nih.gov/clinvar/, My Cancer Genome https://www.mycancergenome.org/) and following the recommendations of accepted guidelines [2,3,4,5,6,7,8,9,10,11,12]. Synonymous mutations not falling in a splicing site or well-known SNPs were considered “benign” mutations. Other mutations without well-established diagnostic/prognostic/predictive significance were not considered clinically relevant, regardless of their Polyphen or SIFT (Sorting Intolerant From Tolerant) scores.

CNS/Intracranial tumors: *IDH1* and *IDH2* analysis is routinely requested for all gliomas as indicated by WHO guidelines [3]. *BRAF* analysis is routinely requested for samples in which a diagnosis of pilocytic astrocytoma, glioneuronal tumors, or pleomorphic astrocytoma (PXA) is being considered [3,13]. *H3F3A* analysis is routinely requested for diffuse midline gliomas [3]. In non-adenomatous lesions of the sellar region, *CTNNB1* and *BRAF* genes are evaluated to discriminate between Rathke cleft cyst, papillary, or adamantine craniopharyngiomas [14].

NSCLCs: *EGFR* and *KRAS* genes are routinely analyzed to evaluate tumor sensitivity to EGFR-TKIs (tyrosin-kinase inhibitors), and mutations reported according to NSCLC molecular testing guidelines [10]. *MET* mutational analysis is also routinely performed according to Lee et al. [15].

CRCs: *KRAS* and *NRAS* mutational analysis is routinely performed to evaluate sensitivity to anti-EGFR monoclonal antibody treatment [16]. According to integrated care pathways currently effective at the Bologna Medical Center, *BRAF* status is evaluated: (i) for prognosis; (ii) in samples where microsatellite instability is performed to distinguish sporadic cases from those that develop in the context of Lynch syndrome [16].

Thyroid nodules: Molecular analysis of *BRAF*, *KRAS*, *HRAS*, *NRAS* is routinely performed according to integrated care pathways of the Bologna Medical Center for preoperative diagnosis on fine needle aspiration specimens and to characterize tumors of follicular cell derivation [2,17]. *TP53* gene is tested when a diagnosis of anaplastic or poorly differentiated thyroid carcinoma is being considered [2,18]. *EIF1AX*, *MED12*, *MET*, *PIK3CA*, *PTEN*, and *TERT* target regions are also analyzed for diagnostic/prognostic purposes [2,17,18,19,20,21,22,23]. Somatic *RET* gene mutations leading to protein constitutive activation are evaluated in medullary carcinomas according to Wells et al. [11].

Melanomas: *BRAF*, *NRAS*, and *c-Kit* are routinely tested to select patients for molecular therapy [6,7].

Pancreatic lesions: *KRAS*, *GNAS*, *RNF43*, and *SMAD4* are analyzed as an adjunct to the preoperative diagnosis of solid and cystic pancreatic tumors [5,24,25].

Other tumors: *TP53* gene is analyzed in selected squamous oral carcinomas following integrated care pathways in place at the Bologna Medical Center; *c-Kit*, *PDGFRα*, and *BRAF* target regions are analyzed in GIST samples following established guidelines [8,9].

### 2.3. Pre-Analytical Evaluation

Two to four unstained 10-μm-thick sections were cut from each selected block, followed by one Hematoxylin and Eosin (H&E) control slide. The tumor/lesional area was marked on the control slide and material for sequence analysis was manually dissected under microscopic guidance from the corresponding 10 μm sections using a sterile blade. For each sample, the proportion of neoplastic/lesional cells vs. non-neoplastic/non-lesional cells in the area marked on the slide and used for DNA extraction was estimated by a pathologist after microscopic evaluation to assess the total number of cells and tumor cell enrichment (i.e., neoplastic cells/total number of cells %). A similar microscopic evaluation was performed to evaluate total cellularity and tumor cell enrichment in cytology smears from fine needle aspiration (FNA). These are routinely microphotographed for archival documentation prior to the scraping of diagnostic material for molecular analysis. Evaluation of total cellularity and tumor cell enrichment is not possible for those fluid samples that have been directly submitted from the clinicians after rinsing the syringe in nucleic acid preserving medium or after dedicated aspiration passes for molecular analysis following FNA (direct fluid cytology samples).

### 2.4. DNA Extraction and Next Generation Sequencing

FFPE samples were extracted using the QuickExtract FFPE DNA Extraction Kit (Epicentre, Madison, WI, USA). DNA from cytological specimens was extracted using the MasterPure DNA Purification Kit (Epicentre, Madison, WI, USA), according to manufacturer’s instruction.

DNA was quantified using Qubit dsDNA BR Assay Kit (Thermo Fisher Scientific, Waltham, MA, USA). Libraries were set up using the Truseq^®^ custom amplicon low input Library Prep Kit (Illumina Inc., San Diego, CA, USA). The samples were then sequenced using a MiSeq sequencing platform (Illumina Inc., San Diego, CA, USA), according to manufacturers’ instruction. Samples used for panel validation (see “Custom-designed multi-gene panel analytical sensitivity” and “Custom-designed multi-gene panel analytical validation” paragraphs) were also analyzed: (i) following a “single-gene approach” using the 454 GS-Junior sequencer (Roche Diagnostics, Rotkreuz, Switzerland) as previously described [26,27,28,29]; (ii) running the same panel using the GeneStudio S5 sequencing platform with the “Ion Ampliseq Library Kit Plus” (Thermo Fisher Scientific, Waltham, MA, USA), according to manufacturer’s instructions.

The sequences obtained were analyzed using VariantStudio Software (Illumina Inc., San Diego, CA, USA) and the Integrative Genomics Viewer 2.3 (IGV) tool (http://software.broadinstitute.org/software/igv/). Only mutations present in at least 5% of the total number of reads analyzed and observed in both strands were considered for mutational calls (see “Custom-designed multi-gene panel analytical sensitivity” and “Custom-designed multi-gene panel analytical validation” paragraphs).

## 3. Results

### 3.1. Statistical Measures of Performance

**Figure 2:**
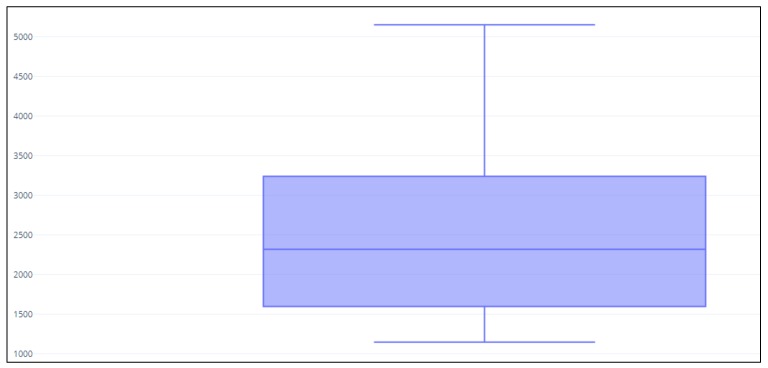
Median next generation sequencing (NGS) read distribution for the entire cohort (1695 cases). *Y*-axis: number of reads.

Using a medium size cartridge (e.g., the v2 cartridge for the MiSeq sequencing platform) and loading from 32 to 40 samples per run we achieved a median coverage of 2500× (Figure 2). An amplicon was considered assessable when at least 200 reads were obtained. The median coverage for each gene is reported in Supplementary Results (Supplementary Table S1 and Supplementary Figure S1).

**Figure 3:**
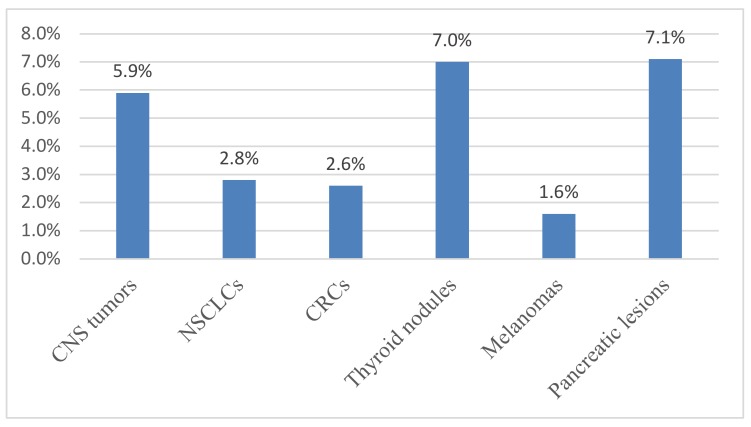
Percentage of cases with no amplifiable DNA. *Y*-axis: frequency of not-amplifiable samples; *X*-axis: Type of lesions analyzed. CNS: central nervous system; NSCLCs: non-small cell lung carcinomas; CRCs: colorectal carcinomas.

No amplifiable DNA (NA-DNA) was obtained from 84 of 1695 specimens (5.0%) due to low quality/quantity DNA. Most specimens of NA-DNA were direct fluid cytology samples from thyroid nodules and pancreatic lesions, with very low amounts of input DNA (less than 5 ng/µL) (Table 1, Figure 3).

### 3.2. Custom-Designed Multi-Gene Panel Analytical Sensitivity

The analytical sensitivity of our custom-designed multi-gene panel was 5%. We tested analytical sensitivity by serially diluting (1:1—100%, 1:2—50%, 1:4—25%, 1:10—10%, 1:20—5%, 1:40—2.5%, 1:100—1%, 1:1000—0.1%) DNA from a pool of samples harboring a homozygous nucleotide polymorphism G>A at the 2470 position of the *EGFR* sequence (c.2470G>A, p.Q787Q) in a pool of samples that did not harbor the nucleotide substitution. Each analysis was repeated three times.

The G>A nucleotide polymorphism was detected in all three runs performed down to a 1:20 (5%) dilution. At 1:40 (2.5%) and 1:100 (1%) dilutions the polymorphism was identified in two of three runs and in one of three runs, respectively.

The same 5% cut-off was obtained by analyzing deletions (i.e., *EGFR* p.E746_A750del, c.2235_2249del) and missense point mutations (i.e., *EGFR* c.2369C>T p.T790M, *KRAS* c.35G>A p.G12D, *KRAS* c.38G>A p.G13D, *NRAS* c.182A>T p.Q61L, *BRAF* c.1799T>A p.V600E, *BRAF* c.1798_1799GT>AA p.V600K, and *PIK3CA* c.3140A>G p.H1047R) using reference tumor DNA in standard paraffin embedded formats (Horizon Diagnostics [HDx], Cambridge, UK) as recently reported by our group in ring trial studies [30,31].

### 3.3. Custom-Designed Multi-Gene Panel Analytical Validation

To evaluate the accuracy of our custom-designed multi-gene panel, we tested 83 samples of the 1695 cases with two orthogonal (alternative) sequencing methods: 53 were analyzed using both the 454 GS-Junior sequencer and our multi-gene panel on the MiSeq platform; 30 samples were analyzed using the custom-designed multi-gene panel on both MiSeq and GeneStudio S5 platforms. To evaluate the precision (reproducibility) of our multi-gene panel, 20 samples were analyzed in two different runs on the same MiSeq platform.

In the 53 samples analyzed with the 454 GS-Junior sequencer and our multi-gene panel on the MiSeq platform, multi-gene custom panel results were concordant with those obtained by a single-gene approach with the 454 GS-Junior sequencer (Table 1).

In the 30 samples where our multi-gene panel was run using both MiSeq and GeneStudio S5 platforms, sequencing results were also concordant, with similar proportions of mutated alleles (Table 2).

The same results were obtained for the 20 samples analyzed in two different runs with the MiSeq platform (Table 3).

### 3.4. Prospective Analysis of Routine Clinical Samples (Clinical Validation)

CNS/Intracranial tumors: The genes most frequently analyzed following the clinician request at the Bologna Medical Center were *IDH1* and *IDH2* (321 samples) (Table 4). A total of 20 CNS samples (5.9%) could not be assessed due to the lack of amplifiable DNA (Table 4, Figure 3). *IDH1* was the biomarker more frequently mutated (22.1%). Among *IDH1/IDH2* mutations, the p.R132H substitution was observed most frequently (76%) (Supplementary Figure S2, Supplementary Table S2). In our cases, 24% of the *IDH1/IDH2* mutated samples harbored mutations different from p.R132H (Supplementary Figure S2, Supplementary Table S2), confirming that the analysis of *IDH1* and *IDH2* genes should not been limited to p.R132H [1,32]. The results of the other genes analyzed (*BRAF*, *H3F3A* and *CTNNB1*) are reported in Table 4.

NSCLCs: *EGFR* and *KRAS* genes were analyzed in 315 NSCLCs. A total of nine NSCLC samples (2.8%) could not be assessed due to the lack of amplifiable DNA (Table 4, Figure 3). *EGFR* was mutated in 14.1% of cases. Deletions in exon 19 were the more frequent alteration (43% of mutated samples) (Supplementary Figure S3, Supplementary Table S2). All cases with the *EGFR* p.T790M also harbored exon 19 deletions or p.L858R mutations. *KRAS* gene was mutated in 38.9% of cases and p.G12C was the most frequent alteration (35% of mutated samples), consistent with what was reported in the literature [33,34]. Almost all *KRAS* mutations (96.0%) were in exon 2; the remaining (4.0%) were p.Q61H substitutions in exon 3 (Supplementary Figure S3, Supplementary Table S2). No mutations were detected in *KRAS* exon 4 (Supplementary Figure S3, Supplementary Table S2). No *MET* exon 14 skipping mutations were observed.

Colorectal carcinomas: A total of 306 CRC samples were analyzed and in all of them the clinician requested the analysis of *KRAS* and *NRAS*. Eight CRC samples (2.6%) could not be assessed due to the lack of amplifiable DNA (Table 4, Figure 3). *KRAS* was mutated in 44.8% and *NRAS* in 5.4% of cases (Table 4). The vast majority of mutations (75% of mutated samples) were in *KRAS* exon 2 (Supplementary Figure S4, Supplementary Table S2). *BRAF* analysis was requested in 205 samples and 15.1% harbored a mutation: all were p.V600E substitutions (Supplementary Figure S4, Supplementary Table S2).

Thyroid nodules: A total of 612 samples were analyzed, including pre-operative material (FNAs and direct fluid cytology samples) and surgical specimens. A total of 43 samples (7.0%) could not be assessed due to the lack of amplifiable DNA (Table 4, Figure 3), 39 of which (91%) were direct fluid cytology samples. *BRAF* gene analysis was requested in all samples analyzed and 19.2% of them harbored *BRAF* mutations (Table 4, Supplementary Figure S5, Supplementary Table S2). Almost all *BRAF* mutations (97.0%) were p.V600E; the remaining were p.V600K (2.0%) and p.T599del (1.0%) (Supplementary Figure S5, Supplementary Table S2). *RAS* genes were mutated in 14.7% of samples: *NRAS* in 8.5%, *KRAS* in 3.3%, and *HRAS* in 2.5% (Table 4, Supplementary Figure S5, Supplementary Table S2). *NRAS* p.Q61R was the most frequent *RAS* mutation (44.0% of *RAS* mutated samples) (Supplementary Figure S5, Supplementary Table S2). Analysis of the *TERT* promoter was requested in 123 samples and 9.8 of them were mutated in the C228T (c.-124C>T) or C250T (c.-146C>T) positions (Table 4). The mutation frequency of *PIK3CA*, *TP53*, *EIF1AX*, *MED12*, *PTEN*, and *RET* genes is reported in Table 4.

Melanomas: Genotyping of 64 samples of primary or metastatic melanoma were analyzed (Table 4, Figure 3). *BRAF* was mutated in 40.6% of samples, *NRAS* in 21.4%, and *c-Kit* in 1.8%. *BRAF* p.V600E was the most frequent alteration (49.0% of mutated melanoma samples) (Supplementary Figure S6, Supplementary Table S2). *BRAF* p.V600K was detected in 15% of mutated melanoma samples (Supplementary Figure S6, Supplementary Table S2). *NRAS* p.Q61R was the more frequent *NRAS* mutation (12.8% of mutated melanoma samples) (Supplementary Figure S6, Supplementary Table S2).

Pancreatic lesions: A total of 42 pre-operative samples (FNAs and direct fluid cytology samples) were analyzed. Three (7.1%) of these specimens could not be assessed due to the lack of amplifiable DNA (Table 4, Figure 3), and all were direct fluid cytology samples. *KRAS* was mutated in 42.8% of the specimens; 90% of these *KRAS* mutations were in exon 2, the remaining (10%) in exon 3 (see Supplementary Figure S7, Supplementary Table S2). This result is consistent with data previously reported demonstrating that *KRAS* alterations in pancreatic lesions do not always involve *KRAS* exon 2 [35,36]. *KRAS* p.G12V was the more frequent alteration (37% of all mutations) (Supplementary Figure S7, Supplementary Table S2). *GNAS* was analyzed in six cases, and in two of them the p.R844H mutation was identified (Table 4). Two of the 42 cases analyzed harbored “double mutations”: one had a *KRAS* p.G12D coexisting with a *KRAS* p.G12V substitution, the other harbored both *KRAS* p.G12V and *GNAS* p.R844H mutations. No mutations were found in *RNF43* and *SMAD4* genes (Table 4).

Other tumors: Seven squamous oral carcinomas were analyzed for the *TP53* gene: five (71.4%) harbored a mutation in *TP53*. One of these five samples had two *TP53* substitutions (p.Y236C and p.R283C). A total of six GISTs were analyzed: four (66.7%) harbored a mutation in *c-Kit* and one case in *PDGFRα* (16.6%). No mutations in *BRAF* exon 15 were identified.

The mutational results of the prospective analysis of routine clinical samples is fully consistent with the data reported in the literature [1,4,12,32,34,35,37,38,39] (Table 5).

## 4. Discussion

The traditional single-gene approach is no longer feasible for the molecular evaluation of solid tumors for diagnostic, prognostic, and predictive purposes, which currently requires the analysis of multiple target genes in a given tumor sample.

**Figure 4:**
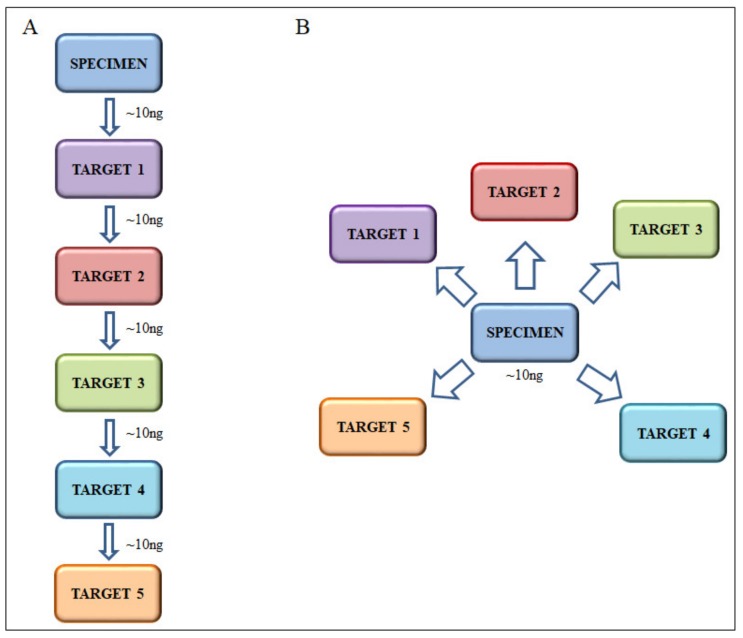
Schemes of molecular analysis using “sequential” (**A**) or “parallel” (**B**) approaches.

Multiple gene targets can be tested by applying in sequence the analysis of several target markers to the same sample using laboratory developed or commercially available kits that are usually based on pyrosequencing or highly sensitive multiplexed mutation-specific real time PCR methods. This approach (the “sequential” approach to molecular analysis) is followed in many molecular pathology laboratories. However, it has several drawbacks: (i) each test has been validated for and requires a definite amount of nuclei acid input (usually ~10 ng of DNA) which can become problematic with limited samples (Figure 4A); (ii) each test has its own cost in terms of both reagent/kit expenses and dedicated technician time so that the total cost of the analysis is determined by the sum of the costs of each individual test; (iii) many tests are tumor marker specific and it is not always possible to analyze different samples for different tumor-specific markers at the same time. An alternative approach (the “parallel” approach to molecular analysis) is based on the utilization of multi-gene NGS cancer panels developed by large medical institutions (e.g., the MSK-IMPACT panel or the SiRe^®^ panel [40,41]) or by companies (e.g., Oncomine Focus Assay (Thermo Fisher Scientific, Waltham, MA, USA)). The advantages of this approach are: (i) with the same amount of nuclei acid input (usually ~10 ng of DNA) a large number of targets can be tested (Figure 4B); (ii) costs are optimized and the cost of the analysis of a given target gene amounts to the total cost divided by the number of genes in the panel; (iii) panels include gene targets common to different tumor types.

An additional advantage of multi-gene NGS panels is that the sequences of genes not initially requested by the clinician remain in laboratory databases. This allows for easily recovering data in case of necessity (e.g., update of guidelines, novel predictive/prognostic markers) without re-extracting DNA and re-sequencing of the specimen. Such repeat analyses would be hard to perform in those samples with low amounts of biological material, such as cytology or biopsy specimens or brain stereotactic biopsies.

We here report the validation of a laboratory developed custom-designed multi-gene NGS panel. Our multi-gene custom panel—designed for the NGS MiSeq platform (Illumina)—has an analytical sensitivity of 5%. Sequencing results are concordant with those obtained using a previously validated single-gene targeted approach using the 454 GS-Junior sequencer [26,27,35] and with those obtained with the Gene Studio S5 platform.

The frequency of mutations in the genes is consistent with that reported in the literature for *IDH1* and *IDH2* in brain tumors [1,32], *EGFR* and *KRAS* in NSCLCs [1,4,12,34,37], *KRAS* and *NRAS* in CRCs [1,4], *BRAF* [37,38,42], *RAS*, *PIK3CA*, and *TERT* in thyroid nodules [1,18,39,42], *BRAF, NRAS* and *cKIT* in melanoma [1,4], and *KRAS* in pancreatic pre-operative samples [1,25,35]. Sequencing results are comparable with those obtained with commercial panels (SiRe panel (Genedin, Rome, Italy), Cancer Hotspot Panel v2 (Thermo Fisher Scientific, Waltham, MA, USA), GeneRead QIAact Lung Panel (Qiagen, Hilden, Germany), Oncomine Solid Tumor Assay (Thermo Fisher Scientific, Waltham, MA, USA), TruSight Tumor 15 (Illumina Inc., San Diego, CA, USA), TruSight Tumor 26 (Illumina Inc., San Diego, CA, USA), 56-gene hotspot panel (Diatech Pharmacogenetics, Jesi (AN), Italy)) [30,31].

Our custom-designed multi-gene NGS panel is reliable, with a 5% overall percentage of non-assessable cases due to lack of amplifiable DNA. In fact, most of the samples that failed due to the lack of amplifiable DNA were cytology samples directly submitted from the clinicians from the pancreas and the thyroid gland for which pre-analytical evaluation of cellularity and tumor cell enrichment were not possible.

Setting up laboratory-developed, custom-designed multi-gene panels for NGS allows for selecting appropriate targets and customizing the proportion between the number of targets/number of samples/sequencing coverage to the specific needs of a given biomedical community. We have engineered a custom-designed multi-gene panel to cover the relevant genes—the analysis of which is required by guidelines and considered standard of care for diagnostic/prognostic/predictive purposes—in tumor types frequently treated in most medical centers. Thus, we have been able to combine the analysis of different types of tumors in the same run. This versatility is not possible with commercially available multi-gene panels that are dedicated to the in-depth analysis of specific tumors, while commercially available comprehensive multi-gene panels include a very large number of targets which limits the number of samples that can be analyzed in the same run (or considerably decreases the coverage of sequencing). Markers useful for therapeutic decision not included in this panel are *BRCA1* and *BRCA2*, important predictive indicators for breast, ovarian, and gastric cancers. However, sequencing of *BRCA1* and *BRCA2* requires the analysis of a number of amplicons—too large to be added to a panel like ours—designed to cover relevant markers for the largest possible number of tumor types following the needs of our medical center. In the Bologna Medical center, *BRCA1* and *BRCA2* are currently analyzed with dedicated panels. Indeed, a general advantage of custom-designed panels is that they can be easily adapted to the needs of specific biomedical communities.

The theoretical number of samples analyzable in one NGS run depends on the following parameters: number of amplicons, total number of theoretical reads available in the run (i.e., the “capacity” of cartridge/chip), and desired target coverage. With our custom-designed multi-gene panel running 32 samples with a 256 amplicons panel on a MiSeq v2 cartridge (~12–15 million of passed reads), the theoretical coverage is ~1800×. In the routine practice, our multi-gene panel allows us to analyze 32–40 samples in the same run with a median coverage of 2500×, using a medium size cartridges (e.g., v2 cartridge for MiSeq sequencer) or chips (e.g., 530 chip for GeneStudio S5).

We designed our panel to analyze the target hot spots of genes relevant for CNS tumors, NSCLCs, CRCs, thyroid nodules, melanomas, pancreatic lesions, oral squamous carcinomas, and GISTs according to updated guidelines and diagnostic/prognostic/predictive clinical needs [2,3,5,6,7,8,9,10,11,14,15,16,17,18,19,24,43].

Since our panel is designed for the relevant gene targets of the tumor types mentioned above, it can be used to analyze different tumor types batched together in a single run, which optimizes turn-around time and costs of NGS. The optimized selection of the genes and the possibility of analyzing for those that are relevant to different tumor types in a given run allows for a higher number of samples to be genotyped per run compared with other NGS multi-gene panels (Table 6).

By analyzing 32–40 samples per run, the mean turnaround time for the 1695 samples was 8.7 working days. The cost was 165–195 € per sample depending on the number of specimens loaded in a given run (March 2020), but independent of the platform used (Illumina or ThermoFisher, using the v2 cartridge or the 530 chip, respectively).

An additional advantage of our custom-designed multi-gene panel is its flexibility: the panel can be rapidly modified at any time, following the discovery of novel new biomarkers or guideline updates independent of the choice and of the timing of commercial diagnostic outfits.

In conclusion, this study reports the validation of a custom-designed multi-gene panel capable of analyzing relevant gene targets—22 oncogenes/oncosuppressor genes—in a variety of solid tumors including those for which genotyping is most frequently required for diagnostic/prognostic/predictive clinical purposes. Our panel is highly sensitive (5% analytical sensitivity) with robust gene coverage, has a high throughput and is highly cost effective.

## Figures and Tables

**Table 1:** Custom-designed multi-gene panel analytical validation of 53 samples using MiSeq and 454 GS-Junior sequencer.

Gene Tested (Number of Samples) (Total Samples: *n* = 53)	454 GS-Junior Results	MiSeq Results
*KRAS* (*n* = 19)	3 *KRAS* p.G12D	3 *KRAS* p.G12D
	2 *KRAS* p.G13D	2 *KRAS* p.G13D
	1 *KRAS* p.Q61K	1 *KRAS* p.Q61K
	1 *KRAS* p.Q61R	1 *KRAS* p.Q61R
	12 WT	12 WT
*NRAS* (*n* = 11)	2 *NRAS* p.Q61R	2 *NRAS* p.Q61R
	9 WT	9 WT
*BRAF* (*n* = 6)	1 *BRAF* p.V600E	1 *BRAF* p.V600E
	1 *BRAF* p.V600K	1 *BRAF* p.V600K
	4 WT	4 WT
*HRAS* (*n* = 4)	1 *HRAS* p.Q61R	1 *HRAS* p.Q61R
	3 WT	3 WT
*EGFR* (*n* = 6)	1 *EGFR* p.E746_A750delELREA	1 *EGFR* p.E746_A750delELREA
	2 *EGFR* p.L858R	2 *EGFR* p.L858R
	3 WT	3 WT
*IDH1*/*IDH2* (*n* = 5)	3 *IDH1* p.R132H	3 *IDH1* p.R132H
	1 *IDH1* p.R132S	1 *IDH1* p.R132S
	1 WT	1 WT
*c-kit*/*PDGFRa* (*n* = 1)	1 *c-Kit* p.V599D	1 *c-Kit* p.V599D
*RET* (*n* = 1)	1 WT	1 WT

WT: wild-type; del: deletion.

**Table 2:** Custom-designed multi-gene panel analytical validation of 30 samples using MiSeq and GeneStudio S5.

Mutational Status MiSeq	% Mutated Allele	Mutational Status GeneStudio S5	% Mutated Allele
*IDH1* p.R132C	23	*IDH1* p.R132C	24
*KRAS* p.G12S	43	*KRAS* p.G12S	42
*KRAS* p.G12R	14	*KRAS* p.G12R	13
*SMAD4* p.E526K	22	*SMAD4* p.E526K	19
*TP53* p.R175H	31	*TP53* p.R175H	32
*TP53* p.R306Ter	48	*TP53* p.R306Ter	51
*c-Kit* p.W557_K558del	51	*c-Kit* p.W557_K558del	44
*c-Kit* p.A502_Y503dup	41	*c-Kit* p.A502_Y503dup	32
*BRAF* p.V600E	29	*BRAF* p.V600E	23
*EGFR* p.E746_A750delELREA	65	*EGFR* p.E746_A750delELREA	48
*EGFR* E746_S752delELREATSinsV	49	*EGFR* p.E746_S752delELREATSinsV	51
*H3F3A* p.K28M	90	*H3F3A* p.K28M	88
*CTNNB1* p.S37C	35	*CTNNB1* p.S37C	40
*RET* p.E768D	51	*RET* p.E768D	51
16 Samples without mutations	/	16 WT	/

WT: wild-type; del: deletion; dup: duplication; ins: insertion; Ter: stop codon.

**Table 3:** Custom-designed multi-gene panel analytical validation of 20 samples tested in two different runs using the same MiSeq sequencing platform.

Gene Tested (Number of Samples) (Total Samples: *n* = 20)	Results-Analysis 1			Results-Analysis 2		
	Mutational Status	Mutated Reads %	Coverage (Reads) ^	Mutational Status	Mutated Reads %	Coverage (Reads) ^
*BRAF* (*n* = 4)	1 p.V600E	15%	3800×	1 p.V600E	7%	3000×
	1 p.V600E	32%	3400×	1 p.V600E	18%	1400×
	WT	0%	500×	WT	0%	700×
	WT	0%	2600×	WT	0%	3000×
*KRAS*/*NRAS*/*BRAF* (*n* = 2)	WT	0%	3600–4500×	WT	0%	2029–5500×
	WT	0%	1250–2500×	WT	0%	2300–3500×
*BRAF*/*KRAS*/*HRAS*/*NRAS* (*n* = 5)	*KRAS* p.G12V	13%	620×	*KRAS* p.G12V	9%	1000×
	*HRAS* p.Q61R	36%	1100×	*HRAS* p.Q61R	39%	3850×
	*HRAS* p.Q61R	35%	3200×	*HRAS* p.Q61R	27%	700×
	WT	0%	3600–4700×	WT	0%	2300–2560×
	WT	0%	1400–3100×	WT	0%	700–1800×
*EGFR*/*KRAS* (*n* = 4) *	1 *EGFR* p.E746_A750delELREA *	44%	5250×	1 *EGFR* p.E746_A750delELREA*	47%	5900×
	1 EGFR p.T790M *	40%	1050×	1 *EGFR* p.T790M *	34%	900×
	1 *EGFR* p.L858R	39%	550×	1 *EGFR* p.L858R	39%	900×
	WT	0%	1750–5130×	WT	0%	1700–2400×
	WT	0%	800–1100×	WT	0%	600–1200×
*KRAS* (*n* = 2)	*KRAS* p.G12C	58%	550×	*KRAS* p.G12C	49%	500×
	*KRAS* p.G12C	17%	6500×	*KRAS* p.G12C	25%	3000×
*IDH1*/*IDH2* (*n* = 3)	1 *IDH1* p.R132C	11%	2500×	1 *IDH1* p.R132C	23%	4700×
	WT	0%	1500–2200×	WT	0%	1300–2050×
	WT	0%	2300–3000×	WT	0%	1800–3500×

* One sample harbored both *EGFR* p.E746_A750delELREA and *EGFR* p.T790M; ^ The target gene coverage range is reported for wild type samples.

**Table 4:** Results of the prospective analysis of routine clinical samples with the custom-designed multi-gene panel.

Type and Number of Samples	Genes Evaluated (Number of Samples per Gene)	Frequency of Mutation (Number of Samples per Gene)
CNS/intracranial tumors		
Brain neoplasms (*n* = 341)	*IDH1* (321)	22.1
	*IDH2* (321)	1.9
	*H3F3A* (12)	16.7
	*BRAF* (14)	14.3
Sellar lesions (*n* = 2)	*CTNNB1* (2)	100.0
	*BRAF* (2)	/
NA: 20 (5.9%)		
NSCLCs (*n* = 315)	*EGFR* (306)	14.1
	*KRAS* (306)	38.9
	MET (50)	/
NA: 9 (2.8%)		
CRCs (*n* = 306)	*KRAS* (298)	44.6
	*NRAS* (298)	5.4
	*BRAF* (205)	15.1
NA: 8 (2.6%)		
Thyroid nodules (*n* = 612)	*BRAF* (568)	19.2
	*KRAS* (481)	3.3
	*HRAS* (481)	2.5
	*NRAS* (481)	8.5
	*TERT* (123)	9.8
	*PIK3CA* (83)	8.4
	*TP53* (67)	7.5
	*EIF1AX* (67)	/
	*MED12* (67)	/
	*PTEN* (37)	/
	*RET* (9)	55.6
NA: 43 (7.0%)		
Melanomas (*n* = 64)	*BRAF* (63)	41.3
	*NRAS* (56)	21.4
	*c-Kit* (56)	1.8
NA: 1 (1.6%)		
Pancreatic lesions (*n* = 42)	*KRAS* (39)	46.2
	*GNAS* (6)	33.3
	*RNF43* (6)	/
	*SMAD4* (6)	/
NA: 3 (7.1%)		
SOCs (*n* = 7)	*TP53* (7)	71.4
GISTs (*n* = 6)	*c-Kit* (6)	66.7
	*PDFGRα* (6)	16.7
	*BRAF* (6)	/

CNS: central nervous system; NSCLCs: non-small cell lung carcinomas; CRCs: colorectal carcinomas; SOC: squamous oral carcinoma; GIST: gastrointestinal stromal tumors; NA not assessable due to the lack of amplifiable DNA.

**Table 5:** Comparison of the custom-designed multi-gene panel mutational results with literature reference data.

Sample Type	Gene	Frequency in Our Series	Frequency Reported in the Literature	References
Brain neoplasms	*IDH1*	22.1	30–39	[1,32]
	*IDH2*	1.9	1–2.8	[1,32]
NSCLCs	*EGFR*	14.1	13.9–22.2	[4,12]
	*KRAS*	38.9	29.7–40	[4,12,34]
CRCs	*KRAS*	44.6	34–44.2	[1,4]
	*NRAS*	5.4	4–4.5	[1,4]
	*BRAF*	15.1	10–11.2	[1,4]
Thyroid nodules	*BRAF*	19.2	13.7–22 *	[1,37,38]
	*HRAS*	2.5	4	[1,39]
	*KRAS*	3.3	2	[1,39]
	*NRAS*	8.5	8	[1,39]
	*PIK3CA*	8.4	4	[1]
	*TP53*	7.5	11	[1]
	*TERT*	9.8	11	[1]
Melanomas	*BRAF*	41.3	36–44	[1,4]
	*NRAS*	21.4	17–23.4	[1,4]
	*c-Kit*	1.8	6.6–8	[1,4]
Pancreatic lesions	*KRAS*	46.2	~45–55	[1,35]

* Values refer to references that have a distribution of preoperative cytology samples comparable with that of our series. NSCLCs: non-small cell lung carcinomas; CRCs: colorectal carcinomas.

**Table 6:** Comparison of commercial NGS panel with our custom-designed multi-gene panel.

Panel (Manufacturer)	Targets	Type of Starting Material	Minimum Amount of Input DNA (Recommended Yeld)	Multiplatform (i.e., NGS from Different Company)	Samples × run (for at least 500–1000× Coverage)
SOPHiA Solid Tumor Solution (Sophia Genetics)	42	Fresh/Frozen	10–50 ng	Yes (ThermoFisher and Illumina)	12–24 *
		FFPE			
Oncomine Focus Assay (ThermoFisher Scientific)	52 ^	Fresh/Frozen	10 ng	No (IonTorrent)	8–16 *
		FFPE			
Oncomine Comprehensive Assay v3 (ThermoFisher Scientific)	161 ^	Fresh/Frozen	30 ng (10 ng per pool)	No (IonTorrent)	8 °
		FFPE			
Human Actionable Solid Tumor Panel (Qiagen)	22	Fresh/Frozen	10–40 ng (fresh)/40–250 ng (FFPE)	Yes (ThermoFisher and Illumina)	24–32 *
		FFPE			
GeneRead QIAact AIT DNA UMI Panel (Qiagen)	30	Fresh/Frozen	40–160 ng	No (Qiagen)	8
		FFPE			
Myriapod^®^ NGS 56G Onco panel (Diatech)	56	Fresh/Frozen	10–25 ng	Yes (ThermoFisher and Illumina)	8–16 *
		FFPE			
Custom-designed multi-gene panel of this study	22	Fresh/Frozen	10–50 ng	Yes (Illumina and ThermoFisher)	32–40
		FFPE			

^ DNA and RNA analysis; * Depending on platform and chip used; ° Only on Ion 540 Chip; FFPE: formalin-fixed and paraffin-embedded.

## Supplementary Materials

The following are available online at https://www.mdpi.com/2075-4418/10/4/250/s1. Figure S1: Median coverage for gene target regions, Figure S2: Frequency of IDH1 and IDH2 mutations in CNS tumor samples, Figure S3: Frequency of EGFR and KRAS mutations in NSCLC samples, Figure S4: Frequency of KRAS, NRAS and BRAF mutations in CRC samples, Figure S5: Frequency of mutations in Thyroid nodules, Figure S6: Frequency of mutations in Melanoma samples, Figure S7: Frequency of mutations in Pancreatic lesions, Table S1: Median coverage for gene target regions, Table S2: Details of mutations detected in samples analyzed.
