# Supplementary material for: Molecular Diagnostic of Solid Tumor Using a Next Generation Sequencing Custom-Designed Multi-Gene Panel
Source: Diagnostics (Basel). 2020 Apr 23;10(4):250. doi: 10.3390/diagnostics10040250 (PMC7236002; doi:10.3390/diagnostics10040250)
Supplement: Supplementary file 1 [file diagnostics-10-00250-s001.pdf]

SUPPLEMENTARY RESULTS

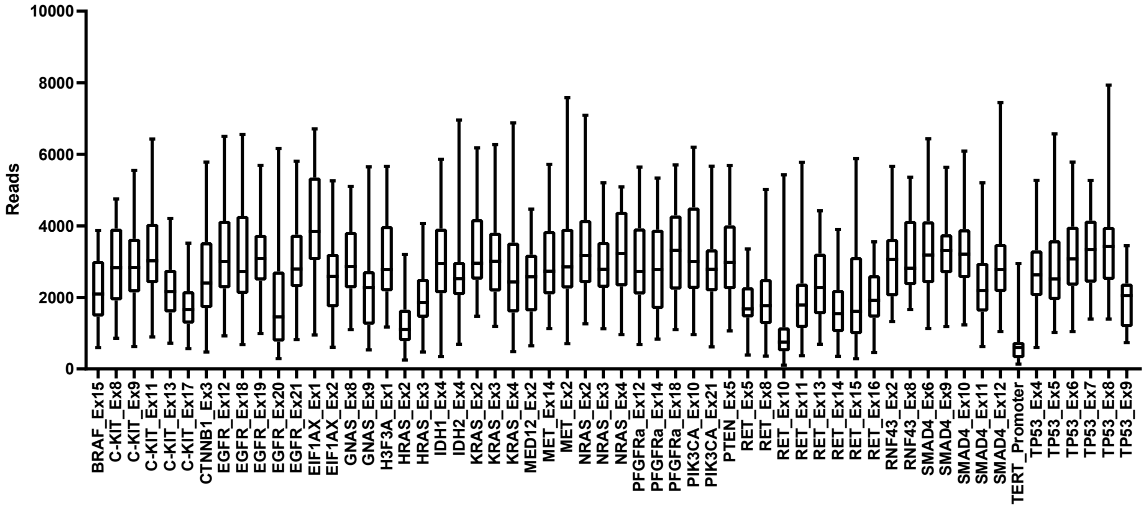

Figure S1. Median coverage for gene target regions. Bars indicate the read coverage range per gene target.

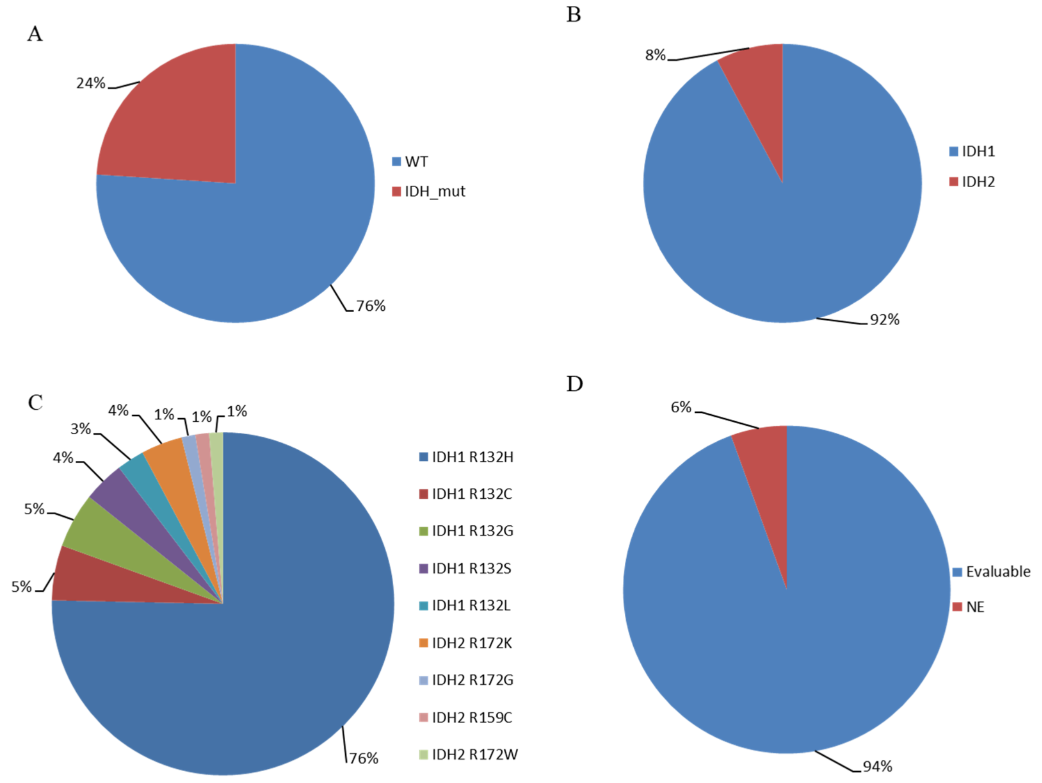

Figure S2. Frequency of *IDH1* and *IDH2* mutations in CNS tumor samples. (**A**) Frequency of *IDH1* or *IDH2* mutations in brain tumor samples; (**B**) Distribution of mutation between *IDH1* and *IDH2* genes; (**C**) Type of *IDH1* or *IDH2* mutations identified; (**D**) Percentage of non-assessable cases due to the lack of amplifiable DNA (NE: not evaluable).

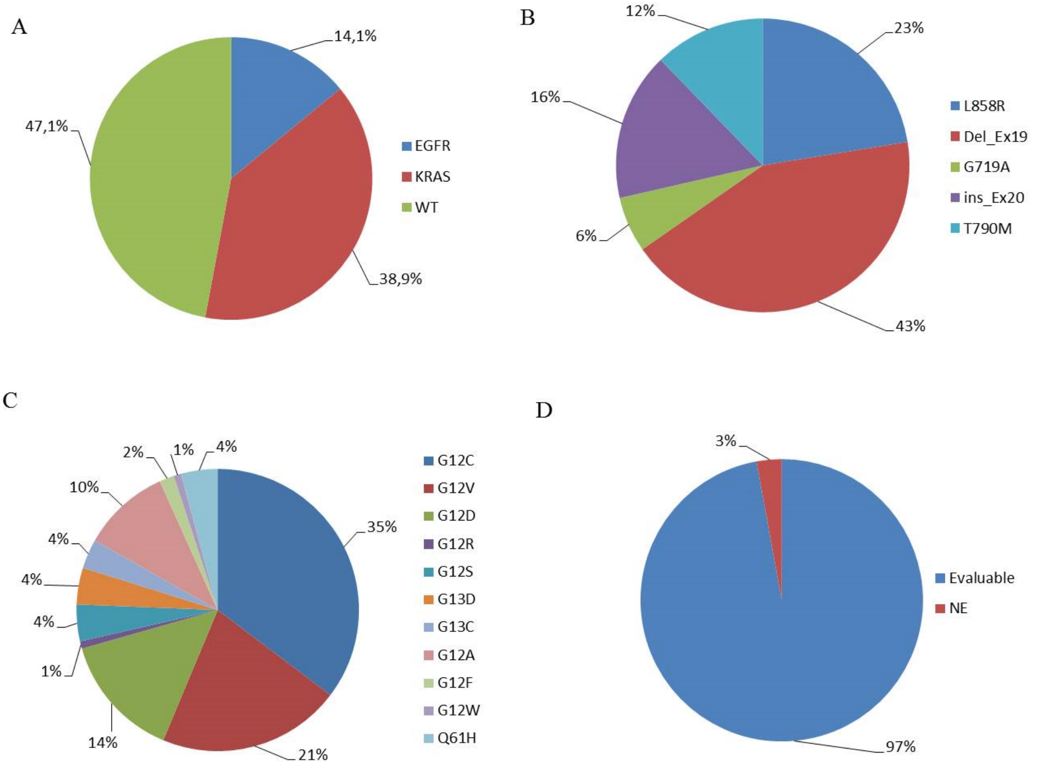

Figure S3. Frequency of *EGFR* and *KRAS* mutations in NSCLC samples. (**A**) Frequency of *EGFR* or *KRAS* mutations in NSCLC samples; (**B**) Type of *EGFR* mutation identified; (**C**) Type of *KRAS* mutations identified; (**D**) Percentage of non-assessable cases due to the lack of amplifiable DNA (NE: not evaluable).

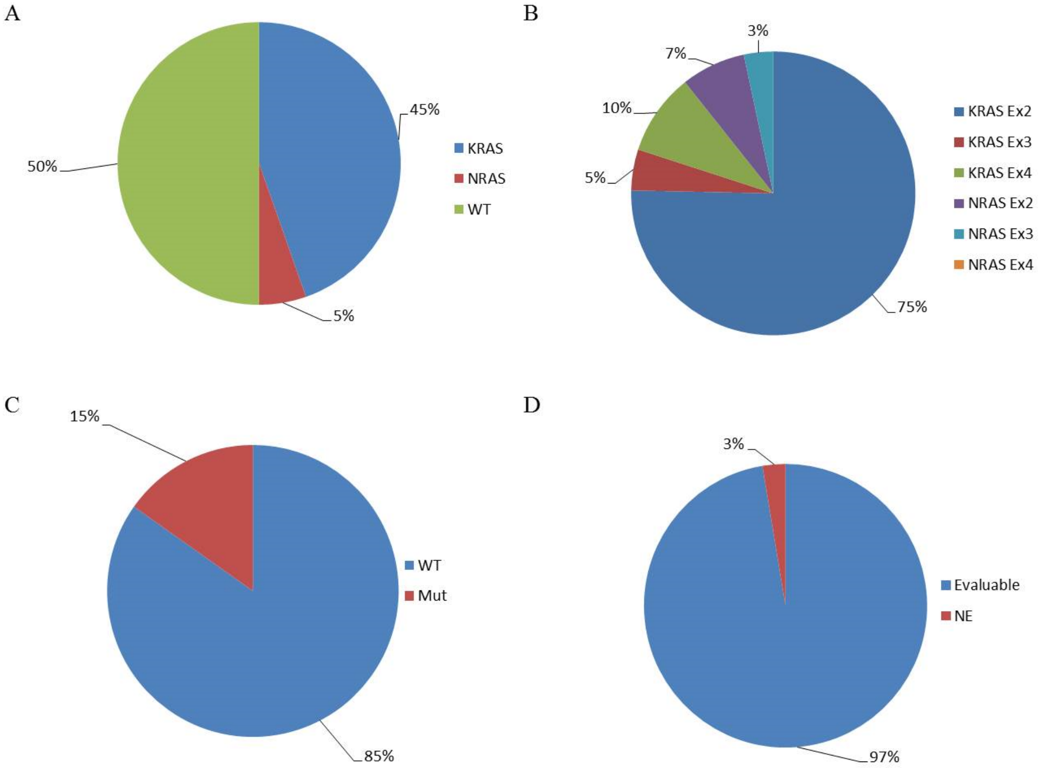

Figure S4. Frequency of *KRAS*, *NRAS* and *BRAF* mutations in CRC samples. (**A**) Frequency of *KRAS* or *NRAS* mutations in CRC samples; (**B**) Type of *KRAS* and *NRAS* mutations identified; (**C**) Frequency of *BRAF* mutations identified (WT: Wild-type; Mut: mutated); (**D**) Percentage of non-assessable cases due to the lack of amplifiable DNA (NE: not evaluable).

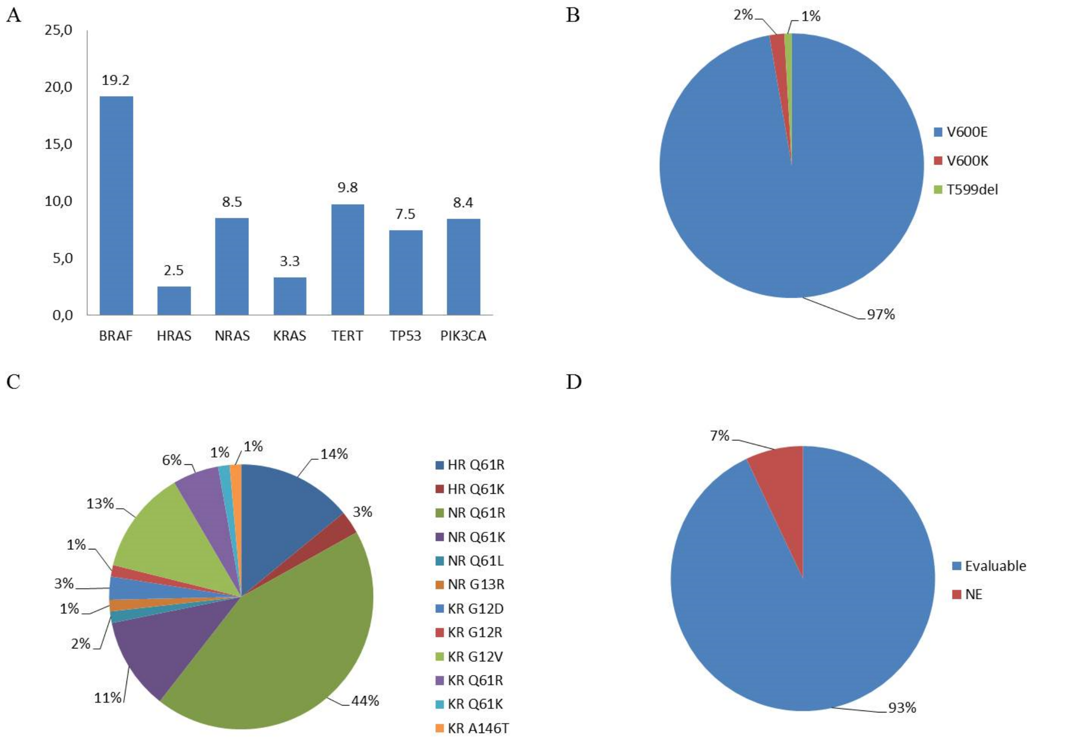

Figure S5. Frequency of mutations in Thyroid nodules. (**A**) Frequency of mutations in thyroid nodules; (**B**) Type of *BRAF* mutations identified; (**C**) Type of *KRAS*, *NRAS* and *HRAS* mutations identified (HR: *HRAS*, NR; *NRAS*, KR: *KRAS*); (**D**) Percentage of non-assessable cases due to the lack of amplifiable DNA (NE: not evaluable).

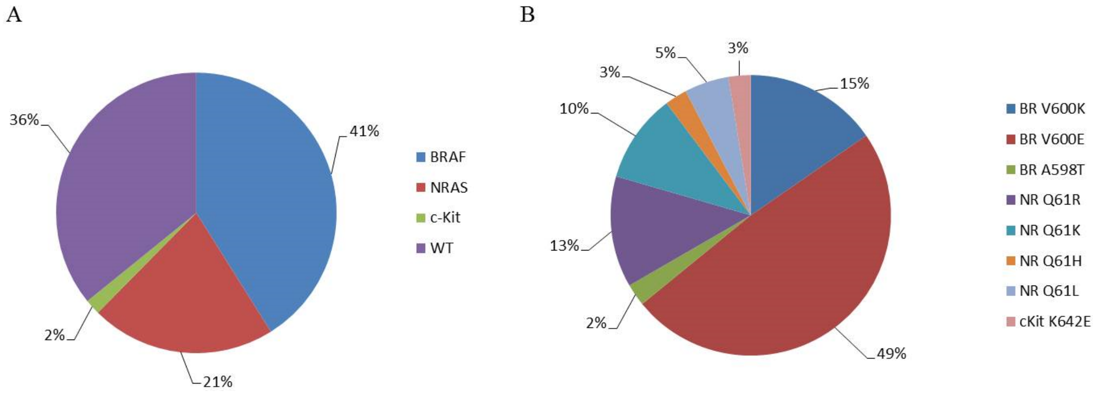

Figure S6. Frequency of mutations in Melanoma samples. (**A**) Frequency of mutations in melanoma samples; (**B**) Type of mutations identified (BR: *BRAF*; NR: *NRAS*).

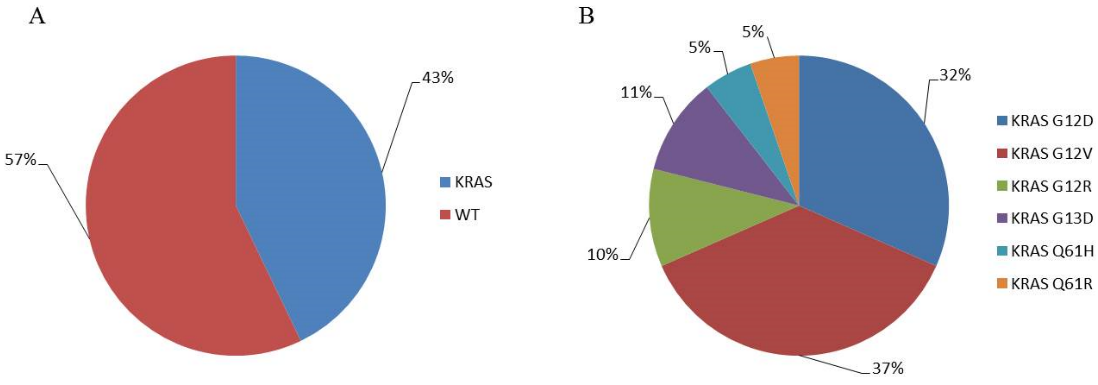

Figure S7. Frequency of mutations in Pancreatic lesions. (**A**) Frequency of *KRAS* mutations in pancreatic lesions; (**B**) Type of *KRAS* mutations identified.

Table S1. Median coverage for gene target regions.

Gene Target Region	Median Coverage (Number of Reads) ^
BRAF Ex15	2237
C-KIT Ex8	2854
C-KIT Ex9	2703–3155
C-KIT Ex11	3090–3359
C-KIT Ex13	2283
C-KIT Ex17	1795–1810
CTNNB1 Ex3	1755–3233
EGFR Ex12	3105–3328
EGFR Ex18	2923–3276
EGFR Ex19	3159
EGFR Ex20	1340–2425
EGFR Ex21	2895–3032
EIF1AX Ex1	4102
EIF1AX Ex2	2571–2673
GNAS Ex8	3006
GNAS Ex9	2258
H3F3A Ex1	3013–3176
HRAS Ex2	1071–1530
HRAS Ex3	1617–2362
IDH1 Ex4	2791–3391
IDH2 Ex4	2332–3073
KRAS Ex2	3197–3467
KRAS Ex3	3157–3198
KRAS Ex4	2283–3188
MED12 Ex2	2450
MET Ex2	2490–3337
MET Ex14	2983–3020
NRAS Ex2	3403
NRAS Ex3	2824–3099
NRAS Ex4	3293–3371
PDGFRα Ex12	2405–3405
PDGFRα Ex14	2884
PDGFRα Ex18	3427–3579
PIK3CA Ex10	3123–3520
PIK3CA Ex21	2175–3297
PTEN Ex5	2939–3137
RET Ex5	1669–1850
RET Ex8	1393–2608
RET Ex10	1110
RET Ex11	1503–2561
RET Ex13	2438
RET Ex14	1464–1913
RET Ex15	1025–3068
RET Ex16	2000
RNF43 Ex2	2158–2912
RNF43 Ex8	1921–2385
SMAD4 Ex6	2943–3466

Table S1. *Cont.*

Gene Target Region	Median Coverage (Number of Reads) ^
SMAD4 Ex9	3253–3368
SMAD4 Ex10	3323–3792
SMAD4 Ex11	1449–2878
SMAD4 Ex12	2743–3173
TERT (g.1295141-g.1295471)	735
TP53 Ex4	2388–3230
TP53 Ex5	2565–3216
TP53 Ex6	3102–3299
TP53 Ex7	3272
TP53 Ex8	3396
TP53 Ex9	1920–3640

^ For gene target regions covered with more than one amplicon the range of median amplicon coverage (minimal read coverage-maximal read coverage) is reported.

Table S2. Details of mutations detected in samples analyzed.

Lesion	Genes Mutated (Number of Mutated Samples)	Type of Mutations (Number of Samples)	Nucleotide Changes
CNS lesions			
Brain neoplasia	*IDH1* (71)	p.R132C (4)	c.394C>T
		p.R132G (4)	c.394G>C
		p.R132H (58)	c.395G>A
		p.R132L (2)	c.395G>T
		p.R132S (3)	c.394G>T
	IDH2 (6)	p.R159C (1)	c.475C>T
		p.R172G (1)	c.514A>G
		p.R172K (3)	c.515G>A
		p.R172W (1)	c.514T>A
	H3F3A (2)	p.K28M (2)	c.83A>T
	BRAF (2)	p.V600E (2)	c.1799T>A
Sellar lesions	*CTNNB1* (2)	p.S37C (2)	c.110C>G

Table S2. *Cont.*

Lesion	Genes Mutated (Number of Mutated Samples)	Type of Mutations (Number of Samples)	Nucleotide Changes
NSCLCs	*EGFR* (35) ^	p.G719A (3)	c.2156G>C
		p.E746_A750delELREA (15)	c.2235_2249del
		p.E746_T751delELREAT (1)	c.2238_2252del
		p.E746_S752delELREATS (1)	c.2236_2256del
		p.L747_E749delLRE (1)	c.2239_2247del
		p.L747_A750delLREA (1)	c.2239_2248del
		p.L747_P753delinsS (1)	c.2240_2257del
		p.S752_I759delSPKANKEI (1)	c.2253_2276del
		p.M766_A767insASV (3)	c.2296_2297ins9
		p.P772_H773insHV (5)	c.2316_2317ins6
		p.T790M (6)	c.2369C>T
		p.L858R (11)	c.2573T>G
	KRAS (119)	p.G12A (12)	c.35G>C
		p.G12C (42)	c.34G>T
		p.G12D (17)	c.35G>A
		p.G12F (2)	c.34_35delinsTT
		p.G12R (1)	c.34G>C
		p.G12S (5)	c.34G>A
		p.G12V (25)	c.35G>T
		p.G12W (1)	c.34_36delinsTGG
		p.G13C (4)	c.37G>T
		p.G13D (5)	c.38G>A
		p.Q61H (5)	c.183A>C
CRC §	*KRAS* (135)	p.G12A (1)	c.35G>C
		p.G12C (9)	c.34G>T
		p.G12D (39)	c.35G>A
		p.G12F (1)	c.34_35delinsTT
		p.G12R (1)	c.34G>C
		p.G12S (7)	c.34G>A
		p.G12V (24)	c.35G>T
		p.G13C (2)	c.37G>T
		p.G13D (25)	c.38G>A
		p.G13R (3)	c.37G>C
		p.Q61H (4)	c.183A>C (3)
			c.183A>T (1)
		p.Q61K (1)	c.181C>A
		p.Q61R (2)	c.182A>G
		p.K117N (3)	c.351A>T
		p.A146P (1)	c.436G>C
		p.A146T (9)	c.436G>A
		p.A146V (1)	c.437G>A
	NRAS (17)	p.G12A (1)	c.35G>C
		p.G12C (1)	c.34G>T
		p.G12D (5)	c.35G>A
		p.G12L (1)	c.34delinsCT
		p.G12V (1)	c.35G>T
		p.G13D (1)	c.38G>A
		p.G13R (1)	c.37C>G
		p.G13V (1)	c.38G>T
		p.Q61K (3)	c.181C>A
		p.Q61L (1)	c.182A>T
		p.Q61R (1)	c.182A>G
	BRAF (31)	p.D594G (1)	c.1781A>G
		p.V600E (30)	c.1799T>A

Table S2. *Cont.*

Lesion	Genes Mutated (Number of Mutated Samples)	Type of Mutations (Number of Samples)	Nucleotide Changes
Thyroid nodules *	*BRAF* (110)	p.T599del (1)	c.1795_1797delACA
		p.V600E (107)	c.1799T>A
		p.V600K (2)	c.1798_1799delinsAA
	KRAS (18)	p.G12D (2)	c.35G>A
		p.G12R (1)	c.34G>C
		p.G12V (9)	c.35G>T
		p.Q61R (4)	c.182A>G
		p.Q61K (1)	c.181C>A
		p.A146T (1)	c.436G>A
	HRAS (12)	p.Q61K (2)	c.181C>A
		p.Q61R (10)	c.182A>G
	NRAS (41)	p.G13R (1)	c.37C>G
		p.Q61K (8)	c.181C>A
		p.Q61L (1)	c.182A>T
		p.Q61R (31)	c.182A>G
	TERT (12)	C228T (12)	c.-124C>T
	PIK3CA (7)	p.R524K (5)	c.1571G>A
		p.P539R (1)	c.1616C>G
		p.E545K (1)	c.1633G>A
	TP53 (5)	p.P67fs (1)	c.200del
		p.C141Y (1)	c.422G>A
		p.R248Q (2)	c.743G>A
		p.R248W (1)	c.742C>T
	RET (5)	p.E768D (1)	c.2304G>C
		p.M918T (4)	c.2753T>C
Melanomas	*BRAF* (26)	p.A598T (1)	c.1792G>A
		p.V600E (19)	c.1799T>A
		p.V600K (6)	c.1798_1799delinsAA
	NRAS (12)	p.Q61H (1)	c.183A>C
		p.Q61K (4)	c.181C>A
		p.Q61L (2)	c.182A>T
		p.Q61R (5)	c.182A>G
	c-Kit (1)	p.K642E (1)	c.1924A>G
Pancreatic lesions	*KRAS* (18)	p.G12D (5)	c.35G>A
		p.G12D+p.G12V (1)	c.35G>A + c.35G>T
		p.G12R (2)	c.34G>C
		p.G12V (6)	c.35G>T
		p.G13D (2)	c.38G>A
		p.Q61H (1)	c.183A>C
		p.Q61R (1)	c.182A>G
	GNAS (2)	p.R844H (2)	c.2531G>A
SOC	*TP53* (5)	p.R175H (1)	c.524G>A
		p.C176Y (1)	c.527C>T
		p.Y236C (1)	c.707A>G
		p.R273H+p.R283C (1)	c.818G>A + c.847C>T
		p.P278L (1)	c.833C>T
GIST	*c-Kit* (4)	p.W557_K558del (1)	c.1669_1674del
		p.V599D (1)	c.1676T>A
		p.V560D (2)	c.1679T>A
	PDFGRα (1)	p.I843_D846delIMHD (1)	c.2527_2538del

^ *EGFR* p.T790M and Ex 20 insertions are found together with *EGFR* ex19 deletions or p.L858R mutation; § One sample was mutated both in *KRAS* and *NRAS* genes; ° One sample was mutated both in *KRAS* and *GNAS* genes; * *TERT*, *PIK3CA* and *TP53* mutations in thyroid nodules were found combined with *BRAF* or *RAS* mutations.
